# Proteomic Analysis of a NAP1 *Clostridium difficile* Clinical Isolate Resistant to Metronidazole

**DOI:** 10.1371/journal.pone.0082622

**Published:** 2014-01-06

**Authors:** Patrick M. Chong, Tarah Lynch, Stuart McCorrister, Pamela Kibsey, Mark Miller, Denise Gravel, Garrett R. Westmacott, Michael R. Mulvey, the Canadian Nosocomial Infection Surveillance Program (CNISP)

**Affiliations:** 1 Public Health Agency of Canada, Winnipeg, Manitoba, Canada; 2 Victoria General Hospital, Victoria, British Columbia, Canada; 3 Jewish General Hospital, Montreal, Quebec, Canada; Institut National de la Recherche Agronomique, France

## Abstract

**Background:**

*Clostridium difficile* is an anaerobic, Gram-positive bacterium that has been implicated as the leading cause of antibiotic-associated diarrhea. Metronidazole is currently the first-line treatment for mild to moderate *C. difficile* infections. Our laboratory isolated a strain of *C. difficile* with a stable resistance phenotype to metronidazole. A shotgun proteomics approach was used to compare differences in the proteomes of metronidazole-resistant and -susceptible isolates.

**Methodology/Principal Findings:**

NAP1 *C. difficile* strains CD26A54_R (Met-resistant), CD26A54_S (reduced- susceptibility), and VLOO13 (Met-susceptible) were grown to mid-log phase, and spiked with metronidazole at concentrations 2 doubling dilutions below the MIC. Peptides from each sample were labeled with iTRAQ and subjected to 2D-LC-MS/MS analysis. In the absence of metronidazole, higher expression was observed of some proteins in *C. difficile* strains CD26A54_S and CD26A54_R that may be involved with reduced susceptibility or resistance to metronidazole, including DNA repair proteins, putative nitroreductases, and the ferric uptake regulator (Fur). After treatment with metronidazole, moderate increases were seen in the expression of stress-related proteins in all strains. A moderate increase was also observed in the expression of the DNA repair protein RecA in CD26A54_R.

**Conclusions/Significance:**

This study provided an in-depth proteomic analysis of a stable, metronidazole-resistant *C. difficile* isolate. The results suggested that a multi-factorial response may be associated with high level metronidazole-resistance in *C. difficile*, including the possible roles of altered iron metabolism and/or DNA repair.

## Introduction

The Gram-positive, spore-forming anaerobe *Clostridium difficile* is the primary etiological agent of hospital-acquired, infectious diarrhea, as well as other associated gastrointestinal diseases, collectively referred to as *C. difficile* infection (CDI). Over 500,000 cases of CDI are reported annually in the US alone, with annual health cost expenditures approaching US$1 billion [1], [2]. While prolonged hospitalization, age (>65), antibiotic treatment or immunosuppressive procedures/diseases are the principal risk factors for the onset of CDI [3]–[6], incidences of community-acquired CDI are also increasing, with infections in young individuals without a history of antibiotic therapy or hospitalization [7]–[9].

Recent trends have shown an increase in the frequency and severity of CDI, as well as an increase in the rates of *C. difficile*-related mortality and recurrence after treatment in North America and Europe; this has been attributed to the emergence of the NAP1/027 strains (North American pulse-field type 1, PCR ribotype 027) [4], [8], [10]–[13]. The NAP1 strain produces higher levels of toxins TcdA (16-fold) and TcdB (23-fold) compared to toxigenic *C. difficile* isolates, and also produce toxin CDT, a clostridial binary toxin encoded by the 6.2-kb CDT locus [14], [15]. NAP1/027 strains display greater resistance to antibiotics, and also possess the capacity to hypersporulate [11], [16], [17].

Metronidazole and vancomycin are the current drugs of choice for treatment of CDI, with metronidazole used as a first-line therapy due to its lower cost, and lower potential of selection for vancomycin-resistant Enterococci [18]–[20]. Recent reports have identified treatment failure and relapse post metronidazole therapy, as well as reduced susceptible or metronidazole-resistant (Met^R^) *C. difficile* strains from clinical isolates, with MIC_Met_ values of 8 to 32 µg/ml [21]–[28]. Metronidazole heteroresistant *C. difficile* strains have also been reported [18], [29]–[31].

Multiple metronidazole resistance mechanisms have been described in *B. fragilis*, including: the seven *nim* genes (*nim* A,B,C,D,E,F, and G), which encode 5-nitroimidazole reductases [32]–[36]; overexpression of RecA, a DNA repair/recombination protein [37]; and disruption of various oxidation/reduction processes and electron transfer reactions [38]. Mechanisms have also been described in *H. pylori* that potentially contribute to metronidazole resistance, such as mutations in the *rdxA* (NADPH-dependent nitroreductase) and *frxA* (NAD(P)H flavin oxidoreductase) genes [39]–[41], changes in activity of non-*rdxA* encoded nitroreductases [42], mutations within the ferric uptake regulator (*fur*) gene [43], [44], overexpression of RecA [45], [46], and overexpression of the *hefA* bacterial efflux pump [47].

In 2009, our laboratory isolated a NAP1/027 *C. difficile* with resistance to metronidazole (initial Etest® MIC >32 µg/ml). As with other reports mentioned above, the MIC to metronidazole of this strain (now called CD26A54_S) decreased to MIC (Etest®) = 2–3 µg/ml upon storage at −80°C; however, when the same strain was continually passaged on agar containing sublethal concentrations of metronidazole, without freezing, the strain became stably resistant to metronidazole; this strain was named CD26A54_R (average MIC = 32 µg/ml) [48]. It should be noted that the MIC values determined by ETest for these strains may be higher when tested using agar dilution or agar incorporation methods, as reported by Moura *et al.* 2013 [49]. The whole genome sequencing of the Met^R^ strain CD26A54_R and the metronidazole-sensitive (Met^S^) strain CD26A54_S has already been described [48]. In this current study, we present a proteomic characterization of the CD26A54_S/R isolates and determine the effect of metronidazole on the proteome of these isolates, focusing on the expression of proteins and processes in *C. difficile* that have been characterized in the literature to be associated with metronidazole-resistance in other bacteria.

## Materials and Methods

### Bacterial strains and growth conditions

Isolation of the CD26A54_S and *C. difficile* CD26A54_R strains was previously described by Lynch *et al.* [48]. The Met^S^ NAP1 *C. difficile* strain VLOO13 was used as a control strain in the proteomics experiments. VLOO13 has an indistinguishable NAP1 macrorestriction pattern and toxin genotype to CD26A54_S and CD26A54_R (data not shown). VLOO13 was kindly provided by Dr. T. Louie (University of Calgary, Calgary, Canada).

For the proteomics iTRAQ analysis, all experiments were performed on isolates that had been passaged three times on Brucella agar supplemented with 10 µg/ml vitamin K, 5 µg/ml hemin, 5% laked sheep blood (BAK+5% LSB, Becton Dickinson, Mississauga, ON, Canada). All strains were grown to perform four biological replicate iTRAQ experiments. Cultures were grown at 37°C in an anaerobic chamber (Coy Laboratory Products Inc, Grass Lake, MI).

Minimal media used for the supplementation experiments was performed using *C. difficile* agar base (OXOID, Nepean, ON, Canada). Colonies were resuspended and normalized to a standard turbidity (McFarland 0.5) in sterile saline, and 200 µl was swabbed over the agar plate. Sterile 5 mm filter disks were then placed on top of the inoculated agar, and spotted with one of the following supplements (stock concentration in (), mg/ml): L-cysteine HCl (50); vitamin K (10); L-homoserine (50); thiamine pyrophosphate (0.1); casamino acids (7%); fetal bovine serum (undiluted); laked sheep blood (undiluted); FeCl_2_ (1); Hemin (7). All plates were incubated for 48 hours prior to analysis.

### Proteomics analysis

**Figure 1:**
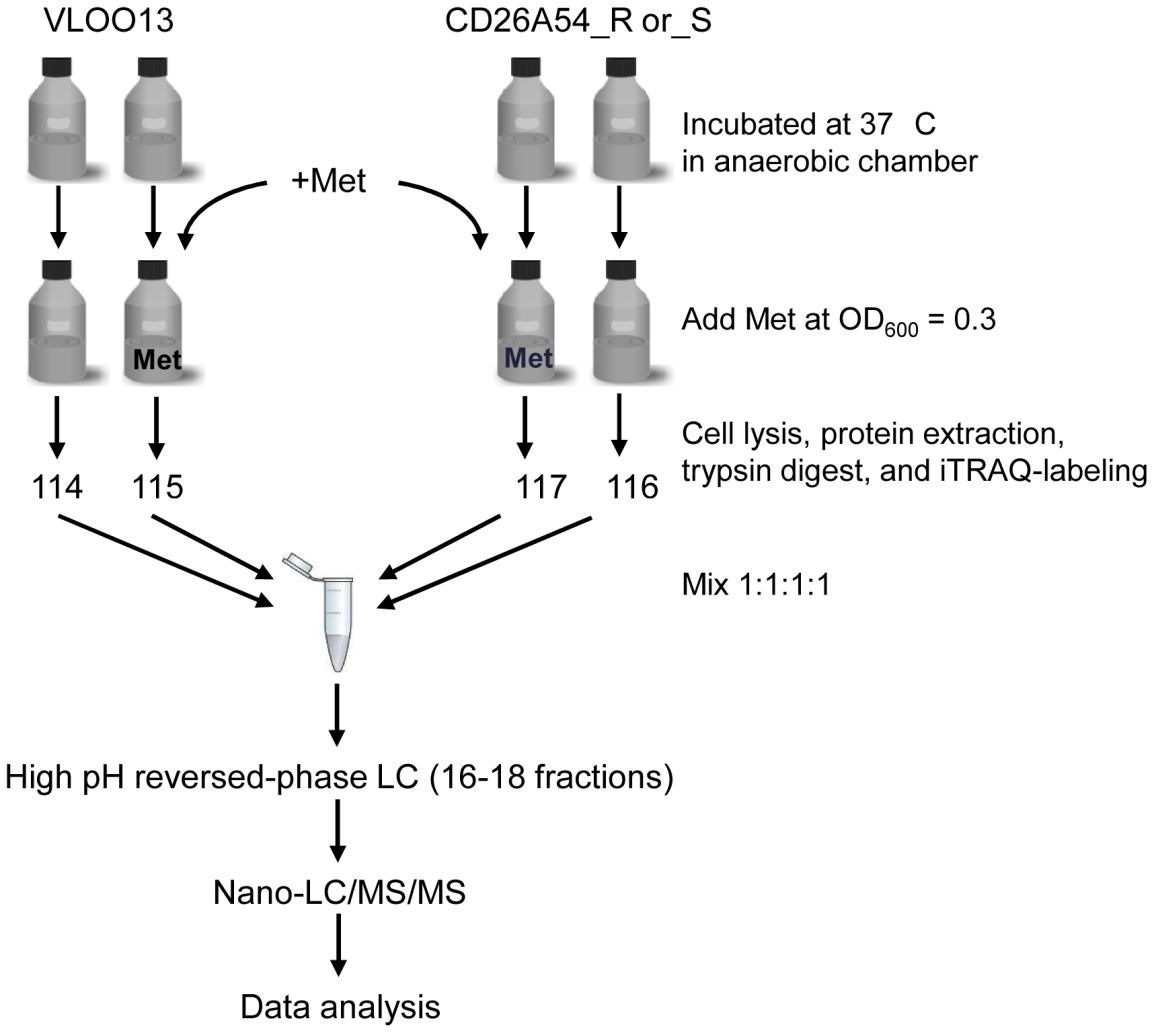
Work flow for digestion and analysis of iTRAQ-labeled peptides from NAP1 *C. difficile* strains. Met, metronidazole. See text for details.

A workflow summarizing the steps leading up to the proteomics analysis of protein extracted from the various strains is shown in Figure 1. A loopful of culture from the BAK+5% LSB plates containing VLOO13, CD26A54_S, or CD26A54_R was inoculated into 5 ml of pre-reduced BHI broth (Becton Dickinson, Mississauga, ON, Canada), and incubated overnight. These cultures were then used to inoculate 200 ml of pre-reduced BHI. Growth (OD_600_) was monitored using an Eppendorf BioPhotometer (ThermoFisher Scientific, San Jose, CA, USA). The *C. difficile* cultures were grown to mid-log phase (OD_600_ between 0.3–0.35), at which point metronidazole was added to the cultures at concentrations 2 doubling dilutions below the MIC_Met_ as follows: CD26A54_R, 4 µg/ml; CD26A54_S, 0.75 µg/ml; and VLOO13, 0.19 µg/ml. While the MIC_Met_ for CD26A54_R, as determined by ETest® on solid agar, was >32 µg/ml, we found that CD26A54_R was not viable in the broth with 8 µg/ml metronidazole, but was still viable at 4 µg/ml metronidazole. In order to observe the immediate effects of metronidazole on the proteome of each strain, all cultures were incubated for an additional 30 minutes prior to cell harvest. Each culture was pelleted (5 min, 15000 rpm, 4°C), and washed twice with cold sterile Milli-Q water. The pellet was then resuspended in 1 ml of Milli-Q-water, centrifuged for 2 minutes at 15,000 rpm, 4°C, and the supernatant removed. 0.1 g cell pellets were stored at −80°C.

### Cell lysis, protein digestion, and iTRAQ-labeling

Thawed cell pellets were homogenized in sterile Milli-Q H_2_O, mixed with 100 µl of 0.1 mm glass beads (Scientific Industries Inc., Bohemia, NY, USA), heated for 5 minutes at 95°C, and vortexed vigorously for 3 min, followed by centrifugation and removal of the supernatant into a 15 ml conical tube. Cold sterile Milli-Q water was added to the beads, followed by vortexing/centrifugation to wash the beads and to extract protein (total of 6 wash/extraction steps); the supernatant from each step was pooled together, mixed thoroughly, and stored at −80°C.

Protein was quantified using a Bichinchoninic Acid (BCA) Protein Assay Kit, with bovine serum albumin (BSA) as standard (Pierce Protein Research Products, Thermo Fisher Scientific). A total of 100 µg of protein from each sample was used for each digestion/labeling reaction.

Protein digestion and iTRAQ-labeling with 4-plex kits were performed according to manufacturer's recommended protocol (AB Sciex, Framingham, MA, USA), and the protein was digested using 5 µg of Trypsin Gold (Promega, Madison, WI, USA). Four separate, independent sets of iTRAQ experiments were performed. Each iTRAQ experiment was comprised of two sets: set 1, VLOO13 and CD26A54_S, +/− metronidazole; set 2, VLOO13 and CD26A54_R, +/− metronidazole In set 1, the VLOO13 control culture and metronidazole-treated culture were labeled with tags 114 and 115, respectively, while the control and metronidazole-spiked CD26A54_S cultures were labeled with tags 116 and 117, respectively. The second set (set 2) consisted of the VLOO13 control (114) and metronidazole-treated (115) cultures (from set 1), along with the control and metronidazole-spiked CD26A54_R cultures that were labeled as described for CD26A54_S.

After final mixing with 1∶1∶1∶1 for each label, the samples were vacuum-dried, rehydrated using offline-LC buffer A (20 mM ammonium formate, pH 10), and stored at −20°C. Both sets of iTRAQ experiments (for CD26A54_S and _R) included four biological-replicates each.

### Offline high-pH reversed-phase fractionation

Samples were resolved with high pH reversed-phase liquid chromatography using an off-line Agilent 1200 series system (Agilent Technologies, Santa Clara, USA) with a Waters guard column (2.1×10 mm) and a Waters XBridge C18 analytical column (3.5 µm, 2.1×100 mm) (Waters Corporation, Milford, MA, USA). The peptides were resolved using a 3–60% mobile phase buffer B (20 mM ammonium formate, 90% acetonitrile) gradient over 75 minutes, at a flow rate of 150 µl/min. 16–18 fractions were collected across the elution profile, concentrated, and then dissolved in nanoLC buffer A (2% acetonitrile, 0.1% formic acid). Samples were stored at −20°C prior to tandem mass spectrometry analysis.

### Nano-LC-MS/MS analysis

Each fraction was analysed using a nano-flow Easy nLC II connected in-line to an LTQ Orbitrap Velos mass spectrometer with a nanoelectrospray ion source at 2.3 kV (ThermoFisher Scientific). The peptide fractions were loaded (10 µl) onto a 0.1×20 mm C_18_-reversed phase trap column (5 µm ReproSil, Dr. Maisch) with nanoLC buffer A, and resolved using a linear gradient of 0–30% nanoLC buffer B (98% acetonitrile, 0.1% formic acid) on a 0.075×100 mm C_18_-reversed phase analytical column (3 µm ReproSil, Dr. Maisch), over 120 min, and flow rate of 300 nl/min. Data was acquired using a data-dependent method, dynamically choosing the top 10 abundant precursor ions from each survey scan for isolation in the LTQ (1.2 *m/z* isolation width), and fragmentation by HCD (higher energy collisional dissociation, 45% normalized collision energy, with 0.1 ms activation time).

### Data processing and statistical analysis

All spectra were processed using Mascot Distiller v2.4.2, and database searching was performed with Mascot v2.3 (Matrix Science). Data were searched against an in-house built, redundant database consisting of protein sequences from completed bacterial genomes of all species from NCBI, downloaded as of October 11, 2012 [ftp.ncbi.nlm.nih.gov/genomes/Bacteria]. Mascot search results were imported and processed using Scaffold Q+ v3.6 (Proteome Software) with X!Tandem, and filtered according to the following parameters: using 80% confidence for peptides, 99% confidences for proteins, and at least 2 peptides per protein. As neither VLOO13 nor CD26A54_S/R had been sequenced, NAP1 *C. difficile* strain R20291 (NCBI Accession number NC_013316) was used as the reference for protein annotation. All four biological replicate iTRAQ experiments were imported into a single Scaffold file. VLOO13 was used as the reference strain for differential protein analysis, in the absence of metronidazole, during analysis of CD26A54_R and CD26A54_S. For analysis after spiking with metronidazole, each strain was analysed separately, using the respective non-treated control strain as reference. In all cases, median values are used, and data was normalized using Scaffold's intensity-based normalization, with reference values averaged across all experiments. Statistical analysis was performed using the Permutations algorithm in Scaffold Q+ v3.6. Only proteins identified at least 3 times out of 4 independent growth experiments, with p<0.05 after correction using the Benjamini-Hochberg method in Microsoft Excel, were considered for further analysis. Statistical analysis was also verified using the Scaffold-calculated log_2_ ratio values that were exported into Perseus and analyzed *via* bi-directional T-test, with Benjamini-Hochberg correction.

Hierarchical clustering analysis was performed using Perseus (MaxQuant, v1.11, Martinsried, Germany), with the following settings: Row, Column distance calculated using the Euclidean algorithm; Row, Column linkage – Complete. Proteins were selected for analysis using Benjamini-Hochberg-corrected p-values (p<0.05), calculated using the Permutations algorithm in Scaffold Q+ v3.6, and clustered according to fold-change.

## Results and Discussion

Lynch *et al.* 2013 [48] previously reported that CD26A5_R, CD26A54_S, and VLOO13 were indistinguishable using pulsed-field gel electrophoresis (NAP1, pattern 001) and contained the same toxin profiles as determined using PCR. However, whole genome sequencing provided some possible insights into the phenotypic variation between strains, including the striking differences observed with the level of metronidazole resistance between the isolates. In an attempt to identify proteins involved in metronidazole-resistant *C. difficile* that may be associated with the resistance phenotype, we employed an iTRAQ-based proteomics approach to study the proteomes of VLOO13, CD26A54_S and CD26A54_R.

### Hierarchical clustering analysis

After combining the results of 4 independent growth experiments, a total of 1942 unique proteins were identified using Scaffold Q+ v.3.6. The protein false discovery rate (FDR) was 0.4%, while the peptide FDR was 0.1%. Relative to VLOO13, and in the absence of metronidazole, 349 (207 increased expression (up), 142 decreased expression (down)) and 433 (235 up, 188 down) differentially expressed proteins were observed in CD26A54_S and CD26A54_R, respectively (p<0.05, 1.5-fold-change) (Table S1). Modest changes were observed to the proteome of CD26A54_S and CD26A54_R after addition of metronidazole, with differential protein expression generally being less than 2-fold relative to the respective non-treated control culture (Table S2). After metronidazole treatment, 40 (29 up, 11 down) proteins were differentially expressed in CD26A54_S, while 32 (28 up, 4 down) proteins were differentially expressed in CD26A54_R (p<0.05, 1.5-fold-change); for VLOO13, 47 proteins (39 up, 8 down) were differentially expressed (Table S2). With a 1.3-fold-change cutoff, 91 and 60 proteins were differentially expressed in CD26A54_S and CD26A54_R after treatment with metronidazole, respectively, with 152 differentially expressed proteins in VLOO13 (Table S2).

**Figure 2:**
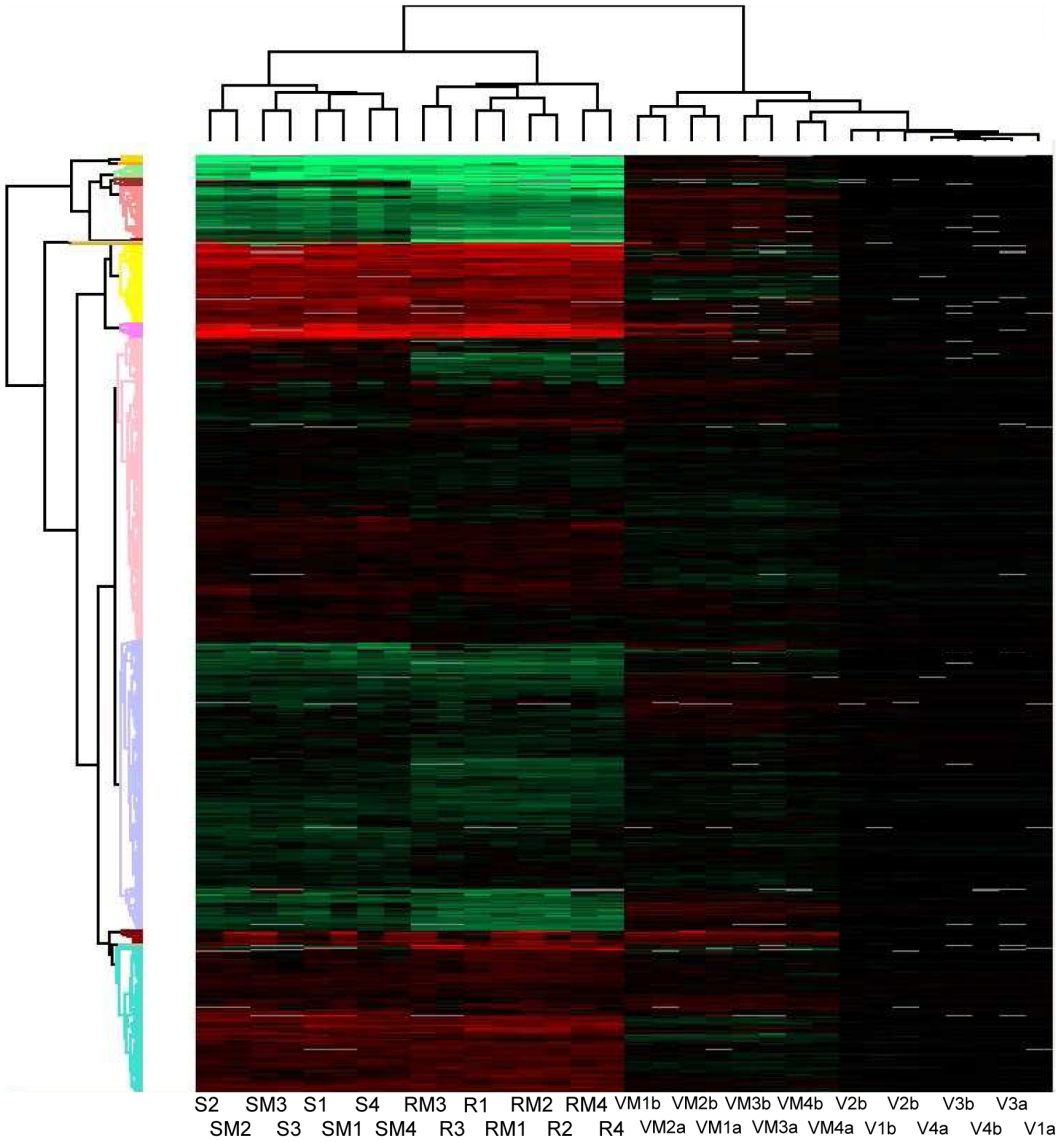
Hierarchical clustering analysis of protein expression using Perseus (Max Quant, v1.1.1.34). V (VLOO13). **VM** (VLOO13+Met). **R** (CD26A54_R). **RM** (CD26A54_R+Met). **S** (CD26A54_S). **SM** (CD26A54_S+Met). Green, reduced expression relative to VLOO13. Red, increased expression relative to VLOO13. Black, no difference in expression. White, no data. Number refers to experiment #. **a**, CD26A54_S set. **b**, CD26A54_R set.

Proteins were clustered according to fold-change in expression, using all fold-change values with p<0.05, as visualized in Figure 2. Analysis revealed VLOO13 was distinct from the CD26A54_S and CD26A54_R strains. Similarly, CD26A54_S and CD26A54_R were also distinct from each other, and were resolved into two separate clusters on the same branch (Fig. 2). In the absence of metronidazole, there were numerous differences in the level of expression of various proteins observed, including those associated with motility, electron transport, oxidative stress response, metabolism and transport of carbohydrates, nucleotides and amino acids, DNA repair, and toxin production (Table 1, Table S1). This is consistent with the results of the previously reported genomics analysis, the differences in MICs to metronidazole, and the general growth differences observed between CD26A54_S and CD26A54_R as reported by Lynch *et al.* [48].

**Table 1:** Differential protein expression of CD26A54_S and CD26A54_R compared against reference strain VLOO13, in the absence of metronidazole.

Gene_locus	Gene	CD26A54_S	CD26A54_R	Description:
CDR20291_0043		2.5±0.2	2.4±0.3	thymidylate synthase
CDR20291_0114		−2.7±0.3	−2.5±0.4	ferredoxin
CDR20291_0485	*nfo*	2.6±0.5	3.3±0.6	endonuclease IV
CDR20291_0490		4.1±1.4	3.3±1.6	endonuclease III
CDR20291_0584	*tcdA*	1.1±0.2	−6.0±2.6	toxin A
CDR20291_0631		−2.1±0.5	−2.8±0.5	DNA repair protein
CDR20291_0755	*rbr*	−1.7±0.1	−1.3±0.2	rubrerythrin
CDR20291_0899	*sbcC*	1.0±0.1	−2.2±0.3	exonuclease subunit C
CDR20291_0957		3.3±0.5	4.6±1.3	nitroreductase family protein
CDR20291_0994	*norV*	2.3±0.5	3.5±0.7	anaerobic nitric oxide reductase flavorubredoxin
CDR20291_1127	*fur*	1.3±0.1	2.6±0.4	ferric uptake regulation protein
CDR20291_1182	*exoA*	2.6±0.2	3.0±0.3	exodeoxyribonuclease
CDR20291_1308	*nimB*	3.3±0.1	3.0±0.2	5-nitroimidazole reductase
CDR20291_1327	*feoA1*	1.6*	7.2±1.9*	ferrous iron transport protein A
CDR20291_1328	*feoB1*	2.3*	11.5±5.8	ferrous iron transport protein B
CDR20291_1354		2.6±0.4	4.5±1.0	drug/sodium antiporter
CDR20291_1373		3.0±0.6	3.2±0.5	rubrerythrin
CDR20291_1385		−1.9±0.2	−1.8±0.2	ferredoxin-NADP(+) reductase subunit alpha
CDR20291_1521		5.2±0.9	5.4±1.3	nitric oxide reductase flavoprotein
CDR20291_1548		no values	6.9±3.0	iron compound ABC transporter substrate-binding protein
CDR20291_1588	*trxA1*	3.0±0.2	4.1±0.2	thioredoxin
CDR20291_1589	*trxB1*	5.5±1.2	7.3±4.2	thioredoxin reductase
CDR20291_1716	*bcp*	7.5±1.5	6.8±1.9	thiol peroxidase
CDR20291_1797		2.7±0.6	1.6±0.3*	conjugative transposon DNA recombination protein
CDR20291_2024	*trxB2*	5.4±0.6	5.0±0.9	thioredoxin reductase
CDR20291_2052		2.3±0.5	2.4±0.3	DNA mismatch repair protein
CDR20291_2075		−1.5±0.3	−1.9±0.1	iron-sulfur binding protein
CDR20291_2101		1.6±0.3	2.4±0.6	ferritin
CDR20291_2242	*trxA2*	−9.4±4.1	−15.3±4.2	thioredoxin
CDR20291_2243	*trxB3*	−3.3±0.8	−4.1±2.1	thioredoxin reductase
CDR20291_3105	*prdC*	−1.4±0.1	−2.2±0.2	electron transfer protein
CDR20291_3231	*uvrB*	1.8±0.3	1.7±0.3	excinuclease ABC subunit B
CDR20291_3232	*uvrC*	1.9±0.3	2.0±0.2	excinuclease ABC subunit C
CDR20291_3233	*uvrA*	1.7±0.2	1.4±0.1	excinuclease ABC subunit A
CDR20291_3444		4.7±2.3	4.9±0.4	ferredoxin

Fold-change values, with standard deviations, of selected proteins, with fold changes >±1.5, p<0.05(with Benjamini-Hochberg correction) are presented here. Values are mean of four independent growth experiments. (-) denotes reduced expression in CD26A54_S or CD26A54_R relative to VLOO13.

not significant (p>0.05), included for comparison only.

After addition of metronidazole to a mid-log culture of VLOO13, the protein expression profile was distinct from the non-treated control; however, hierarchical cluster analysis (Fig. 2) cannot distinguish between the metronidazole-treated and control strains of CD26A54_S or CD26A54_R on the basis of differential protein expression.

This would appear to indicate that, under these experimental conditions, treatment with metronidazole does not significantly alter protein expression in CD26A54_S or CD26A54_R, and that some resistance mechanisms may be expressed in the absence of metronidazole. Specific changes to the proteome in these isolates will be discussed in detail below. The focus of this discussion will be on the identification of proteins potentially involved with metrondazole-resistance in *C. difficile*, based on putative resistance mechanisms described in the literature with other bacteria.

### Proteomics analysis

Differential expression of electron transport and redox-active proteins was observed in both CD26A54_S and CD2654_R compared to VLOO13. In the absence of metronidazole, differential expression was observed in both CD26A54_S and CD26A54_R, compared to VLOO13 (Table 1). It has been well established that metronidazole is effective against organisms residing in low-intracellular redox environments [50]. Studies with *B. fragilis*, *H. pylori*, and the anaerobic protoazoan *G. lamblia*, indicate that various proteins involved in electron transfer reactions (i.e. pyruvate ferredoxin/flavodoxin oxidoreductase (PFOR), ferredoxin, hydrogenases, etc) are associated with the reduction of metronidazole to its active form [51]–[54].

Pyruvate oxidation, catalyzed by PFOR, has been linked to reduction of metronidazole; PFOR catalyzes the CoA-dependent decarboxylation of pyruvate to acetyl-CoA, with the electrons transferred to the amine group of metronidazole *via* ferredoxin or flavodoxin [52]. Little-to-no change in the expression of PFOR was observed in CD26A54_R, compared to VLOO13, with or without metronidazole under the experimental conditions (data not shown). In *H. pylori*, a reduction in the activity of pyruvate oxidation activity (i.e. pyruvate oxidoreductase) was observed in Met^R^ and strains [38], [51], [52]. Similarly, Diniz *et al.* [38] reported the downregulation of flavodoxin and PFOR in *B. fragilis* with reduced-susceptibility to metronidazole. Thus, while downregulation of PFOR expression appears to be a factor in the development of metronidazole resistance, this was not observed in the CD26A54_S/R isolates. However, Lynch *et al.* [48] reported a Gly423Glu mutation in the amino acid sequence of PFOR of CD26A54_R, which may change the activity of the protein and lead to decreased activation of metronidazole, without a change in the level of expression of PFOR.

Differential expression of ferredoxin was observed in CD26A54_S/R, in the absence of metronidazole (Table 1). Ferredoxin is an intermediate in the reduction of the pro-drug form of metronidazole to its radical toxic form. Thus, reduced expression of ferredoxin may lead to reduced activation of metronidazole, and possibly result in a resistant or reduced susceptibility phenotype. However, the differential expression data for ferredoxin in CD26A54_S/R is ambiguous, as there are at least two different genes coding for ferredoxin, with one ferredoxin (CDR20291_0114,) being reduced <2.5-fold relative to VLOO13, and the other ferredoxin (CDR20291_3444) increased >4.5-fold relative to VLOO13, in CD26A54_S/R. Therefore, we currently cannot draw any conclusions in metronidazole-resistance with respect to ferredoxin.

Our data also indicated higher expression of other redox-active proteins in both CD26A54_R and CD26A54_S, including putative nitric oxide reductase flavoprotein and putative nitric oxide reductase flavorubredoxin, which are believed to be associated with protection against the effects of nitric oxide (Table 1 [55]). There are also genes encoding other oxidative stress-related proteins, such as thioredoxin, thioredoxin reductase, and rubrerythrin that were differentially expressed in CD26A54_R and CD26A54_S; however, expression was more highly expressed or decreased compared to VLOO13, depending on the gene expressed (Table 1). Further analysis will be required to determine which redox proteins may be involved with metronidazole resistance in CD26A54_R.

Addition of metronidazole did not appear to stimulate differential expression of redox proteins in CD26A54_S or CD26A54_R (data not shown).

Putative nitroimidazole reductase proteins are expressed more highly in CD26A54_R and CD26A54_S compared to VLOO13. In the absence of metronidazole, expression of 5-nitroimidazole reductase, encoded by *nimB*, appears to be expressed at higher levels in the CD26A54_S (∼3-fold) and CD26A54_R (∼3-fold) isolates compared to the Met^S^ reference strain VLOO13 (Table 1). The increased expression is independent of the presence of metronidazole (data not shown). Reduced susceptibility to 5-nitroimidazole drugs has been associated with the presence of a *nim*-encoded nitroimidazole reductase, which converts 4- or 5-nitroimidazole to 4- or 5-aminoimidazole, avoiding the formation of the toxic nitroso radicals [36], [56]. Therefore, the increased expression of 5-nitroimidazole reductase in CD26A54_S and CD26A54_R may be a factor in the reduced susceptibility and resistance to metronidazole observed in these isolates. Inducible metronidazole resistance has also been reported in *nim*-negative strains of *B. fragilis*, and *Prevotella baroniae*, suggesting that mechanisms other than *nim* genes are also involved with metronidazole resistance and adaptation [57]–[60].

A putative nitroreductase family protein was also expressed more highly in CD26A54_S and CD26A54_R, with levels approximately 3- and 4-fold higher relative to VLOO13, respectively (Table 1). It has been speculated by Jorgensen *et al.* [42] that *H. pylori* may contain multiple nitroreductase enzymes that may contribute to metronidazole resistance in this bacterium. It would be interesting to determine whether the nitroreductase family protein in *C. difficile* also contributes to the level of metronidazole resistance observed with CD2654_S or CD26A54_R.

DNA repair proteins are differentially expressed in CD26A54_R and CD26A54_S relative to VLOO13. In the absence of metronidazole, our proteomics analysis identified several putative DNA repair proteins with elevated expression in the CD26A54_S and CD26A54_R strains as compared to the VLOO13 control strain. One such protein is the excinuclease ABC, also referred to as the UvrABC repair system, which is involved in the repair of DNA lesions. Compared to VLOO13, expression of UvrABC appears to be elevated up to ∼2-fold in the CD26A54_S and CD26A54_R strains. Given its role in DNA repair, UvrABC may contribute to the metronidazole resistance of CD26A54_R and reduced susceptibility of CD26A54_S. Other DNA repair mechanisms also appear to be more highly expressed in CD26A54_S/R as compared to VLOO13, including ∼3-fold increases in expression to exodeoxyribonuclease, endonuclease III, endonuclease IV, and DNA mismatch repair protein (∼2-fold increase) (Table 1), possibly explaining the elevated MIC_Met_ in CD26A54_S/R isolates. The activated form of metronidazole primarily interacts with DNA, leading to DNA strand-breakage, and also induces oxidative stress in the cell, both of which lead to subsequent cell death [41], [61]. As such, resistance mechanisms against metronidazole could also include enhanced DNA repair.

After treatment with metronidazole, the expression of DNA repair protein RecA was induced 1.6-fold in CD26A54_R, and approximately 1.2-fold in CD26A54_S and VLOO13 strains (Table 2). RecA is a highly conserved protein that plays a central role in the ATP-dependent maintenance and repair of DNA, through homologous recombination and repair of double-stranded DNA breaks, and is also a central protein in the SOS response [62], [63]. RecA is thought to be part of the adaptive response to metronidazole in *B. fragilis* and *H. pylori* [37], [45]. Overexpression of RecA in *B. fragilis* lead to increased resistance to metronidazole relative to the wild-type and *recA* mutant strains [37]. Introduction of phagemids containing genes from metronidazole-resistant *H. pylori* into metronidazole-sensitive *E. coli* and *H. pylori* induced resistance to the antibiotic [45]. However, the expression of RecA in CD26A54_R after metronidazole treatment is only moderately increased (1.6-fold) relative to CD26A54_S and VLOO13 (1.2-fold); further analysis will be required to determine what role, if any, RecA plays in metronidazole resistance in *C. difficile*. Given that the RecA protein can also play a role in antimicrobial resistance, virulence, as well as general stress response in bacteria [63], it was not surprising to observe that in all of the isolates tested, there was also increased expression of various cellular stress-related proteins after treatment with metronidazole, including the 10 and 60 kDa chaperonins, heat shock protein, and the heat-inducible transcription repressor HrcA (Table 2). The molecular chaperone DnaK, along with co-chaperones GrpE and DnaJ, and the heat shock response protein ClpB were induced upon treatment with metronidazole, suggesting that metronidazole induces a general stress response in *C. difficile*. A comprehensive list of proteins differentially expressed after addition of metronidazole is shown in Table S2.

**Table 2:** Differential protein expression in VLOO13 (V), CD26A54_S (S), and CD26A54_R (R), after spiking of the cultures with metronidazole, at O.D._600_ = 0.3.

Gene locus	Gene	V_Met_/V_c_	S_Met_/S_c_	R_Met_/R_c_	Description:
CDR20291_0194	*groES*	1.6±0.2	1.7±0.2	1.7±0.1	10 kDa chaperonin
CDR20291_0195	*groEL*	1.5±0.1	1.5±0.1	1.5±0.1	60 kDa chaperonin
CDR20291_0899	*sbcC*	not significant	no value	1.4±0.2	exonuclease subunit C
CDR20291_0957		1.5±0.2	1.3±0.1	1.2±0.1	nitroreductase family protein
CDR20291_1169	*recA*	1.2±0.1	1.2±0.1	1.6±0.1	RecA protein (recombinase A)
CDR20291_1328	*feoB1*	not significant	no value	−1.7±0.2	ferrous iron transport protein B
CDR20291_1548		not significant	no value	−2.2±0.3	iron compound ABC transporter substrate binding protein
CDR20291_1933	*clpB*	1.5±0.1	1.8±0.1	1.5±0.1	chaperone
CDR20291_2353	*dnaJ*	1.3±0.1	1.4±0.1	1.6±0.2	chaperone protein DnaJ
CDR20291_2354	*dnaK*	1.4±0.1	1.6±0.1	1.7±0.1	molecular chaperone DnaK
CDR20291_2355	*grpE*	1.5±0.1	1.8±0.1	1.7±0.1	heat shock protein GrpE
CDR20291_2356	*hrcA*	2.0±0.2	2.1±0.1	2.1±0.1	heat-inducible transcription repressor
CDR20291_3233	*uvrA*	1.2±0.1	1.1±0.0	1.5±0.1	excinuclease ABC subunit A
CDR20291_3234	*uvrB*	1.3±0.1	1.3±0.1	1.6±0.1	excinuclease ABC subunit B

Metronidazole-treated strains (Met) were compared against respective non-treated strains (c) strains. Fold-change values of selected proteins, with p<0.05, (with Benjamini-Hochberg correction) are presented here. Values shown are mean of four independent growth experiments. (-) denotes reduced expression of Met-treated sample compared to non-treated control.

With CD26A54_R, there was also a reduction of iron uptake/transport proteins after treatment with metronidazole. Iron compound ABC transporter substrate protein (CDR20291_1548) and ferrous iron transport protein B (*feoB1*, CDR20291_1328) were reduced 2.2- and 1.7-fold, respectively (Table 2). This suggests that iron uptake may be diminished after treatment with metronidazole in the resistant strain. A reduction of iron may result in the synthesis of fewer redox-active proteins, and thusly fewer molecules of activated metronidazole.

Expression of the ferric uptake regulator (Fur) is increased in CD26A54_R compared to CD26A54_S and VLOO13. In the absence of metronidazole, a ∼2.6-fold and ∼1.3-fold increase in the expression of the ferric uptake regulator (Fur) protein was observed in CD26A54_R and CD26A54_S relative to VLOO13, respectively (Table 1); expression of Fur did not change substantially after treatment with metronidazole (data not shown). Fur is a central regulator of iron homeostasis in bacteria, mediating iron-dependent repression of iron uptake systems. Fur can also regulate gene expression in the absence of iron as an apo-protein [64], behaving as a multifunctional regulator of numerous genes, including acid, salt, and oxidative stress, modulation of bacterial virulence, redox metabolism, and colonization. It also auto-regulates its own expression when high iron content leads to repression of *fur* expression [64]–[70].

Genomic analysis of CD26A54_R by Lynch *et al.* [48] indicated the presence of a single nucleotide variation (SNV) in the *fur* gene of CD26A54_R, where a guanine is replaced with an adenine residue at position 1353228 (from R20291 genome), leading to a Glu41Lys mutation; CD26A54_S did not contain any mutation in the *fur* gene. Point mutations to Fur may lead to deregulation of genes that encode iron uptake or transport, even in the presence of iron. As indicated in Table 1, there is a significant increase in expression of ferrous iron transport B (∼12-fold increase) and non-significant (p>0.05) increase in expression of ferrous iron transport A (∼7-fold increase) in CD26A54_R, suggesting a defect in iron uptake and/or regulation.

Point mutations to the *fur* gene have also been shown to increase metronidazole resistance in *H. pylori*. These point mutations to the *fur* gene lead to the expression of a modified Fur protein diminished in its capacity to repress expression of the *sodB* gene, which encodes iron-cofactored superoxide dismutase (SodB) [64]; higher expression of SodB enables *H. pylori* to counteract the oxidative stress generated by activated metronidazole [44]. Superoxide dismutase, encoded by *sodA* in *C. difficile*, was not identified in our proteomics analysis, so it is not clear if this protein is involved in metronidazole resistance in *C. difficile* as in *H. pylori*.

CD26A54_R grows on basal medium only when supplemented with exogenous iron. Growth experiments in BHI broth indicated that CD26A54_R exhibits a longer lag phase compared to CD26A54_S and VLOO13, and also grows to a lower final cell density, while no significant differences in growth observed between CD26A54_S and VLOO13 [48]. When grown on basal *Clostridium difficile* agar, both CD26A54_S and VLOO13 grew similar to what was observed on rich media, while no viable cells were observed for CD26A54_R (Table 3). Addition of amino acids, Vitamin K, or phosphate to filter disks spotted on the surface of the agar did not stimulate growth of CD26A54_R. However, CD26A54_R grew on the basal medium after the addition of 5% laked sheep blood (5% LSB) (Table 3), indicating that components found in blood were required for growth of this strain on basal medium; growth was not observed after addition of fetal bovine serum. Since the presence of a SNV in the *fur* gene may result in a defective Fur protein and impaired iron-related Fur activity in CD26A54_R, we wanted to see if the addition of excess iron to the basal medium would stimulate growth. As with 5% LSB, the addition of FeCl_2_ to the *C. difficile* agar stimulated growth of CD26A54_R, suggesting that the Glu41Lys Fur is defective with respect to iron homeostasis, possibly related to inefficient iron uptake. Addition of hemin, which is also found in blood, alleviated a growth restriction of CD26A54_R (Table 3). This is also consistent with the frameshift mutation found in the *hemN* gene by Lynch *et al.* [48]; *hemN* encodes coproporphyroinogen III oxidase, a component of the heme biosynthesis pathway. Defects in the Fur and coproporphyroinogen III oxidase may explain why CD26A54_R grew more slowly compared to CD26A54_S and VLOO13 in BHI broth and basal *C. difficile* agar. Impairment in hemin biosynthesis or uptake, can lead to defects in electron transport [71], [72]. If electron transport is impaired in CD26A54_R, one consequence may be that fewer molecules of metronidazole are reduced to its active form, allowing for this strain's inherent defenses to counter the lower levels of active metronidazole.

**Table 3:** Supplements that were spotted on 5*Clostridium difficile* base agar.

Supplement	Concentration	Amount (µl)	VLOO13	CD26A54_S	CD26A54_R
L-cysteine HCl	50 mg/ml	20	+	+	−
5% Laked sheep blood	undiluted	20	+	+	+
Vitamin K	10 mg/ml	20	+	+	−
L-homoserine	50 mg/ml	20	+	+	−
Thiamine pyrophosphate	100 µg/ml	20	+	+	−
Fetal bovine serum	undiluted	20	+	+	−
Casamino acids	7%	20	+	+	−
FeCl_2_	1 mg/ml	20	+	+	+
Hemin	5 mg/ml	20	+	+	+

(+), growth. (−), no growth.

Taken together, the results of our proteomic analysis demonstrate that although both strains arose from the same isolate, CD26A54_S and CD26A54_R are divergent; this is consistent with the previously reported genomic analysis results of these isolates, growth curve analysis, as well as the clear differences in level of resistance to metronidazole of each strain [48]. We observed that there are multiple proteins that may contribute to the metronidazole-resistance/reduced susceptible phenotype of CD26A54_R and CD26A54_S, respectively, including a putative 5-nitroimidazole reductase, and various proteins involved with DNA repair and iron metabolism. Interestingly, differential expression of the vast majority of those proteins in the CD26A554_S/R strains was not dependent on the presence of metronidazole. As with reports of other metronidazole-resistant bacteria, expression of multiple enzymes/pathways may be involved in resistance/adaptation to metronidazole. Indeed, studies with other metronidazole-resistant anaerobes point to the multifactorial nature of metronidazole resistance [38], [54]. The proteomic studies reported here have identified proteins that are potentially associated with metronidazole-resistance in *C. difficile*; further studies are required to confirm the physiological roles of these proteins with metronidazole resistance. To our knowledge, this work represents the most comprehensive analysis with a metronidazole-resistant NAP1 *C. difficile* strain to date.

## Supporting Information

Table S1 **Protein expression (fold-change) in CD26A54_S (S) and CD26A54_R (R) relative to VLOO13 (V).** Fold-change values of proteins shown with p<0.05 (Permutations Test, non-corrected) for S and/or R. Values shown are means of four independent growth experiments. (-) denotes reduced expression in S or R, relative to V. SD, standard deviation.
(XLSX)

Table S2 **Differential protein expression in VLOO13 (V), CD26A54_S (S), and CD26A54_R (R) after spiking of the cultures with metronidazole, at O.D._600_ = 0.3.** Metronidazole (Met)-treated strains were compared against respective non-treated control strains (C). Fold-change values of differentially expressed proteins, with 1.3-fold cut-off are presented here. Values shown are means of four independent growth experiments. (-) denotes reduced expression of Met-treated sample compared to non-treated control. SD, standard deviation.
(XLSX)
